# The Hospital Officer's Bookshelf

**Published:** 1912-12-07

**Authors:** 


### THE HOSPITAL OFFICER'S BOOKSHELF.

Fifty Doctors against Alcohol. (Issued under the auspices of the Literature Committee of the National Brotherhood Council.)

The series of addresses, essays, notes, quotations, and other scraps which make up this volume is gathered from the proceedings at a succession of meetings held last year in Birmingham on the occasion of the annual meeting of the British Medical Association in that city. The list of authors includes some of the most earnest and energetic of the advocates in the medical profession of total abstinence; indeed, it is not going too far to say that several of them are well known as "cranks" upon the subject. Others of the fifty are more reasonable sort of people, and less frankly propagandist in their aims. Admitting to the full the grave evils consequent upon alcoholic excess, we cannot feel that the cause of true temperance is much advanced by a volume such as the present one. At the same time, it is right to state that many of the contributions are unexceptionable. Mr. Eccles, to give an instance, sees that the moderate drinker is the man who has to be "converted," and realises that (for the success of total abstinence principles) so far not much impression has been made upon him. We do not think Mr. Eccles presents a very strong case; but at least he does it fairly tactfully and moderately.

Science French Course. By C. W. Paget Moffatt, M.A., M.B., B.C. (London: W. B. Clive, University Tutorial Press, Ltd. 1912. Price 3s. 6d.)

On perusing this very interestingly arranged manual one cannot fail to be struck by the clearness with which the leading points of French grammar are set out, and also by the large amount of syntax the author has been able to explain in an easy manner. We can readily conceive that this book would be of great utility to those whose French has dwindled to microscopic proportions, but who still possess sufficient linguistic intelligence to assimilate rapidly the coarser features of a foreign language. As is indicated by the title, this course is mainly directed to enable students to read scientific books in French, and half the book consists of readings in various branches of science. Naturally, the student of any one particular science will find the course somewhat restricted, but the elements of grammar and construction are so lucidly arranged that he will have no difficulty in proceeding further with ordinary application. Dr. Moffatt's French Course is in no way inferior to his similar venture in the German language, and candidates for the membership of the Royal College of Physicians may well avail themselves of either of these excellent manuals.

Mother and Baby. By Selina F. Fox, M.D. (London: J. and A. Churchill. 1912. Pp. 200. Price 1s. 6d. net.)

There seems no end to the production of books which seek to guide the expectant or actual mother in the principles of infant care and management. This last newcomer in the lists has some evident merits. It is well printed and produced; not too long or technical; sound and practical as to doctrine, as a general rule. Here and there can be found statements to which the critical may object. Thus, the weights given for various stages of infantile development are somewhat on the skimpy side; no doubt a (perfectly healthy) child of two years might weigh 25 lb., but the majority would certainly turn the scale at rather more than this. So, again, it is also true that babies have a habit of cutting their first teeth at six or seven months of age; but Dr. Fox might add that many babies are later than this in commencing their dentition, and that a very few are even earlier. She might also, as a medical practitioner, abandon the traditional tone of puling sentimentality which seems to pervade this class of publication; and she might with advantage edit some of the statements of the patent food proprietors on the advertisement pages. But taken all round her book is a favourable specimen of its class, and one deserving of success.

Ophthalmic Nursing. By Sydney Stephenson, M.B., F.R.C.S.E. Third Edition, revised and enlarged. (London: The Scientific Press, Ltd. Pp. 229. Price 3s. 6d. net.)

It is ten years since the second edition of this book was published, and of course longer still since the first edition. In the interval Mr. Stephenson has "arrived," as author, teacher, and expert ophthalmologist. His book on ophthalmic nursing, though very greatly modified by the changed methods of surgical practice in the meanwhile, remains—as it was from the first—a most valuable and original contribution to the branch of service with which it deals. Ophthalmic nursing is perhaps even more a specialty than any other special department of nursing in general; and a sound text-book by a competent authority is a very great asset to any who are called upon to take it up. Needless to say, a general training should precede the process of specialisation; for the fundamental principles upon which ophthalmic surgery is based are no different from those which underlie surgical practice in other regions of the body. It is pleasing to note that Mr. Stephenson sticks strictly to business, without any of those irritating divagations into sentimentalism and poetry which most women authors on nursing seem unable to resist. Another valuable feature of his book is the lists of instruments needed for various common ophthalmic operations; and the appendix in which these are printed is succeeded by an even more useful one still, where all the instruments in question are pictorially illustrated. A third appendix contains a glossary of the special terms used by ophthalmologists, with explanations of them. In this glossary the author is very guarded in his views as to the origin of chalazion, whereas in the text of chapter vii. he is quite dogmatic on the subject. We recommend his book most cordially to all who have to deal with ophthalmic cases, whether as nurses or surgeons.

Anæsthetics and their Administration. By Sir Frederic W. Hewitt, M.D., M.V.O., Anæsthetist to H.M. the King, late Anæsthetist to King Edward VII., etc.; assisted by Henry Robinson, M.D., Anæsthetist to the Samaritan Hospital and to the Cancer Hospital. Fourth Edition. (London: Macmillan and Co. 1912. Pp. 676. Price 15s. net.)

It must be very exasperating to a medical author to spend infinite time and labour over the compilation of a text-book, only to find within a few months or weeks that some important new development has rendered a good deal of it obsolete, or at least incomplete. This fate was conspicuous in the literature of syphilis, where first the Wassermann reaction and next the salvarsan therapy upset all previous conceptions of diagnosis and treatment. In the department of anæsthetics a similar evolution took place. One author a few years ago stated in a book that anæsthesia by administering ether on an open mask was impossible, only to be confronted the very same year by the importation of the open ether method from America, where, as a matter of fact, it had been flourishing for some time. Even Sir Frederic Hewitt's insight and power of prevision failed to warn him of the advent of lumbar anæsthesia, intravenous ether, hedonal, and many similar novelties of the last three or four years; so that the third edition of his well-known text-book has latterly ceased to reflect the whole of modern practice in his specialty. In bringing up to date his very excellent monograph, a great deal of labour has been entailed; but the task was worth doing, and it has been well done. As the book now stands the new edition takes rank—as the previous ones in their own day did also—as the premier work on anæsthetics in the English language. Its merits are many and conspicuous; its defects remarkably few and comparatively trivial. Two new procedures have been described since the book went to press, but as neither of them seems likely to be of permanent value or interest, it remains true to say that the book is fully up to date in every way. The difference between the present edition and the last one is shown by the fact that, roughly speaking, a third of the book has been re-written, while of the rest hardly a page is unaltered or unamended. Sir Frederic's book has long provided the bases upon which all the shorter text-books have been built up, and the new edition is such that this form of flattery is not likely to be abandoned. We regard this work as one of the best text-books on any medical subject now before the profession.

Cancer of the Breast. By the late Cecil H. Leaf, M.B., F.R.C.S. (London: Constable and Co. 1912. Pp. 154. Price 10s. 6d. net.)

As a conscientious anatomist Mr. Leaf possessed a more intimate knowledge of the lymphatic supply and connections of the breast than perhaps any other operator in this country, and this knowledge he utilised to the full in discussing the problem of the way to eradicate cancer which has attacked that organ. It is, therefore, not surprising to find that Mr. Leaf's chapters on the anatomy of the breast and axilla are thoroughly well done, and that his beautiful drawings are of great value in illustrating the text. As a clinician Mr. Leaf was painstaking and methodical, and his wide reading of foreign authors was another point in his favour. But he was not by temperament either a scientist or a surgeon, and the clinical sections of his book reflect his limitations. The consecutive series of cases tabulated in the ninth and tenth chapters show results which are really good, and should suffice to demonstrate what only "Christian Scientists" and similar irrationals doubt—namely, that early surgical operation affords a reasonably good chance of cure for cancer of the breast, far better than is given by any other remedy or treatment. Mr. Leaf's monograph forms a useful summary of its subject, but the price is much too high and will probably militate against any considerable circulation.

Radium and Radio-Activity. By A. T. Cameron, M.A., B.Sc. (London: Society for Promoting Christian Knowledge. 1912. Pp. 185. Price 2s. 6d.)

In this compact and well-arranged book the author sets forth succinctly the present state of knowledge of radium and radio-activity He is compelled to assume in his readers a slight general knowledge of chemistry and physics; for otherwise he would have to double his pages at the least. In this assumption he is quite justified; for, after all, those who know nothing whatever of elementary chemistry are hardly in a fit state to appreciate the significance of radio-activity. To the medical profession there need be nothing alarming in this, for no more knowledge is presumed than a medical student has to acquire for his preliminary scientific examination. The exposition of the whole subject is lucid and accurate; and when one reflects that every single fact in connection with it has been discovered in the last fourteen years the marvel is not that we know so little, but that we know so much about these extraordinary substances and their properties. Anyone who wishes to get an idea of the progress of chemistry in the last two decades may profitably compare the composition of the atmosphere as given in any text-book of the 'nineties with the imposing analysis printed on the last page of Mr. Cameron's book. After a list of some sixteen constituents, more or less considerable, we come to niton, of which in one million parts of air there exist 0.00000000000006 parts; and thorium emanation, of which there is but 0.000000000000000002!

The First Signs of Insanity: Their Prevention and Treatment. By Bernard Hollander, M.D. (Stanley Paul and Co. Price 10s. 6d.)

The author states that this book, which is an exposition in popular form of his views on insanity, appeals to a wide circle of readers. We presume that this means that he is writing for the general public, or at least for that section of it which is especially interested in this subject. A perusal of its pages tends to corroborate this view. It is hardly in accordance with the strictest code of medical ethics for a professional man to address a book of this character to his *clientèle*. More especially is this so when he directs his readers' attention to other works from his pen, and informs them where a full description of his cures will be found. His method of treating the various subjects may appeal to some lay members of the community. The medical man seeking information and assistance will, in our opinion, be disappointed, but then—the book was not specially intended for him.

British Red Cross Society's Nursing Manual. No. 2. By James Cantlie, F.R.C.S., V.D. (Cassell and Co. 1912. Price 1s. net.)

The industry and ingenuity of Mr. Cantlie are prodigious. In this small Manual, which completes the series (the first and third having already been published) of Elementary Manuals for members of the Voluntary Aid Detachments of the Territorial Force, these qualities are well displayed. Since there are already elementary manuals of home nursing in existence, some of them quite excellent, the task of making up a new one is somewhat difficult. In so restricted a field most of the flowers have already been culled by others; Mr. Cantlie, however, has managed to find some choice blossoms which seem to have been overlooked before, and it is with great pleasure that we feel able to say that his Manual is an improvement on all its predecessors of similar scope. We are not quite sure he has done wisely in including pictures of the local lesions of typhoid fever and smallpox; but as a general rule he steers successfully clear of the pitfall of technicality, and his advice is always full of sound and practical common sense.

Materia Medica and Pharmacy. By R. R. Bennett, B.Sc. Second Edition. 1912. (H. K. Lewis. Pp. 227. Price 4s. 6d. net.)

This short manual is one of those which has rapidly won its way into the affections of the medical student. It does not pretend to be encyclopædic, to contain the whole art of prescribing and dispensing, or the whole science of pharmacology. It cannot take the place of Dr. Hale White's larger work on this subject; but it fills very adequately another, perhaps rather humbler, niche. It is very concise, well arranged, well expressed, and well produced. It contains an enormous store of knowledge useful to the student of medicine, and essential for the successful treatment of patients. By presenting to students this often-neglected subject of their curriculum in the attractive method which Mr. Bennett has adopted, a great deal is done towards stimulating their interest in pharmacology and materia medica. We feel sure that at University College Hospital, where the author is pharmacist and teacher of pharmacy, the students are well instructed in that part of their work; and we trust that the appearance of this new edition is evidence that those at other centres of training are taking advantage of Mr. Bennett's excellent book.

The Human Body. By Arthur Keith, M.D., LL.D. Home University Library. (London: Williams and Norgate. 1912. Price 1s.)

A popular book on the study of man is nowadays almost predestined to be successful, and when such a work is made available in the form of a shilling pocket volume it is not to be wondered at that Dr. Keith's contribution to the Home University Library is already in demand. It is, indeed, creditable to the publishers of this Library that they have been able to secure the services of the Hunterian Professor and the Conservator of the Museum of the Royal College of Surgeons for the purpose of producing this volume. We need do no more than to say that Dr. Keith has handled this always fascinating subject in a popular manner, while providing a useful treatise for those who desire to learn. "Development," "Racial and Sexual Characteristics," "Mechanism," and "Genealogy" are the titles of some of the chapters. Even the examination-scared medical student will find it pleasant and profitable.

On Alcoholism: Its Clinical Aspects and Treatment. By Francis Hare, M.D. (London: J. and A. Churchill. 1912. Pp. 269. Price 5s. net.)

This is a book for which we predict a rapid success. The condition with which it deals is not uncommon, unfortunately, and is so difficult to cure that the general practitioner is driven to his wit's end to know how to deal with it. The ordinary text-books of medicine are much too vague on the subject to be of any practical help; and a monograph by a specialist of Dr. Hare's experience is just what hundreds of doctors have felt the need for. The author is well known through his connection with the Norwood Sanatorium at Beckenham, and his intimate experience of every phase of the inebriate problem illuminates every chapter of the book. This, combined with a very fresh and interesting style of writing and with a sound common-sense outlook, makes the book very easy reading.

Dr. Hare takes a much more optimistic view of the prognosis of a case of chronic alcoholism than that which most general practitioners are accustomed to express. This is quite possibly due to the fact that he sees his patients under the control of sanatorium life and discipline, and away from the home life or other environment which has caused or frustrated their alcoholism; few diseases are so hopeless and heartbreaking to treat as alcoholism in a patient who is still surrounded by the evil influences to which he has succumbed. The last chapter, dealing with the "cure" of alcoholism and the different meanings which can be read into that word, is an excellent summing-up of this aspect of the disease—if, indeed, alcoholism can rightly be called a "disease." The use of strychnine, atropine, and cinchona in combination as a substitute for alcohol has for some time been gaining popularity amongst medical men. Dr. Hare gives the most minute directions for this treatment, directions almost too meticulous to be uniformly applicable. The medical profession is deeply indebted to Dr. Hare for providing such an excellent account of alcoholism, an account so thoroughly pervaded with good sense and clinical wisdom that his promised companion volume upon Morphinism will be cordially welcomed.

A Handbook for Midwives and Maternity Nurses. By Comyns Berkeley, M.D., F.R.C.P. Third and Enlarged Edition. Illustrated. (London: Cassell and Co., Ltd. 1912. Price 5s.)

That this little text-book has already reached its third edition is but the fulfilment of the predictions made at the time of its first appearance. Dr. Comyns Berkeley is no less successful in teaching midwives than in expounding the obstetrical art to his medical students. In many respects this neat little manual is a model; the language is always clear and intelligible, the teaching dogmatic and very much to the point, and the arrangement logical and well sub-divided. Add an excellent index and a series of useful illustrations, and the result is a text-book on the subject of midwifery which is well adapted for those preparing for the examination of the Central Midwives Board. This edition contains an important appendix on cancer of the uterus and two new ones on venereal diseases and Cæsarian section. No detailed comment is now necessary on the subject-matter of the various sections, but without detracting from its merits as a midwives' text-book, we have a decided inclination to recommend Dr. Berkeley's book to the close attention of medical students and even to that of not a few general practitioners.

Mouth Hygiene and Mouth Sepsis. By John Sayre Marshall, M.D., Sc.D. (Philadelphia: J. B. Lippincott Company. 1912. With Plates. Pp. 262. Price 6s. net.)

When an author sets out to write a book that shall suit the requirements of such a mixed circle of readers as he refers to in his preface, it is safe to presume that one or other section will fail to find satisfaction in his pages. Dr. Marshall would have been wiser had he frankly addressed himself to the lay public, the education of whom is the obvious and very laudable purpose of his book. This object, we may fairly observe, would have stood a better chance of success had the author placed a severe check on his verbosity and produced a more succinct essay, published at a much smaller price. Whether or not the remote effects of oral sepsis do include such dire complaints as immorality, drunkenness, and insanity, the fact remains that the subject of oral hygiene is one of vast importance to the individual and to the community. Any endeavour to bring home to parents, teachers, nurses, and others the great benefits which accompany the *dens sanus in ore sano* is surely worthy of support. But it must be confessed that Dr. Marshall's book is open to improvement in general and to correction in many particulars. The author's attempts to explain technical terms are not always happy, and many are left untranslated—in fact, there is much in the book which must be almost unintelligible to the lay reader, and much that will appear platitudinous to the dental surgeon. Those who know can appreciate the author's excellent gospel, but they are not likely to be in the audience to which Dr. Marshall wishes to address himself.

